# Liangxue Tongyu Prescription Alleviates Brain Damage in Acute Intracerebral Hemorrhage Rats by Regulating Intestinal Mucosal Barrier Function

**DOI:** 10.1155/2022/2197763

**Published:** 2022-12-17

**Authors:** Yu Zhou, Zijian Zhang, Yingying Sun, Dandan Zhou, Yang Zhao, Jianxiang Li, Weifeng Guo

**Affiliations:** ^1^First School of Clinical Medicine, Nanjing University of Chinese Medicine, Nanjing 210023, China; ^2^Department of Neurology, Nanjing Hospital of Chinese Medicine Affiliated to Nanjing University of Chinese Medicine, Nanjing 210022, China; ^3^School of Chinese Medicine, School of Integrated Chinese and Western Medicine, Nanjing University of Chinese Medicine, Nanjing 210023, China; ^4^Department of Neurology, Affiliated Hospital of Nanjing University of Chinese Medicine, Nanjing 210029, China

## Abstract

**Background:**

Liangxue Tongyu prescription (LTP) is a commonly used formula for acute intracerebral hemorrhage (AICH) in clinical practice that has significant ameliorative effects on neurological deficits and gastrointestinal dysfunction, yet the mechanism remains elusive. The aim of this study was to investigate the pathway by which LTP alleviates brain damage in AICH rats.

**Methods:**

The AICH rat models were established by autologous caudal arterial blood injection. The neurological function scores were evaluated before and after treatment. The water content and the volume of Evans blue staining in the brain were measured to reflect the degree of brain damage. RT-PCR was used to detect the inflammatory factors of the brain. Western blotting was used to detect the expression of the tight junction proteins zonula occludens 1 (ZO-1), occludin (OCLN), and claudin (CLDN) in the brain and colon, followed by mucin 2 (MUC2), secretory immunoglobulin A (SIgA), and G protein-coupled receptor 43 (GPR43) in the colon. Flow cytometry was used to detect the ratios of helper T cells 17 (Th17) and regulatory T cells (Treg) in peripheral blood, and the vagus nerve (VN) discharge signals were collected.

**Results:**

LTP reduced the brain damage of the AICH rats. Compared with the model group, LTP significantly improved the permeability of the colonic mucosa, promoted the secretion of MUC2, SigA, and GPR43 in the colon, and regulated the immune balance of peripheral T cells. The AICH rats had significantly faster VN discharge rates and lower amplitudes than normal rats, and these abnormalities were corrected in the LTP and probiotics groups.

**Conclusion:**

LTP can effectively reduce the degree of brain damage in AICH rats, and the mechanism may be that it can play a neuroprotective role by regulating the function of the intestinal mucosal barrier.

## 1. Introduction

Due to certain factors, such as rapid onset, long duration, and severe sequelae, stroke poses a considerable burden on public health. The results of the Global Burden of Disease (GBD) 2019 study showed that China has the highest incidence of stroke at over 30%, and intracranial hemorrhage (ICH) accounts for approximately 15% of all strokes [1]. Compared with cerebral infarction (CI), ICH has higher mortality and disability rates [2]. In the acute phase, a hemorrhage mass can directly cause primary brain injury, and the continued existence and expansion of hematoma can lead to secondary brain injury [3]. Currently, conventional treatments that rely on surgery, dehydration to lower intracranial pressure, and blood pressure control have no definite clinical benefit for the prognosis of patients with ICH [4]. Therefore, exploring effective means to improve stroke prognosis and increase survival rates is necessary.

With the increased understanding of the physiopathological process of ICH, mitigation of secondary brain injury has become a therapeutic focus. This is not only related to the inflammatory response, neurotoxicity, blood‒brain barrier (BBB) disruption, and brain edema but Cheng et al. also confirmed that intestinal mucosal structure and function are disrupted in ICH mice [5, 6]. Furthermore, when gut flora homeostasis was improved, neurological deficits were relieved [7]. Based on these findings, the interaction between the gut and the central nervous system (CNS) has received our attention; moreover, the previously reported theory of the gut-brain axis demonstrates that the two can regulate each other through humoral, cellular immune, and neuronal pathways [8]. The intestinal mucosal barrier (IMB) has a complex barrier structure to defend against pathogen colonization and endotoxin invasion, with intestinal epithelial cells (IECs) playing the primary protective role [9]. IECs are interconnected by basolateral membrane apical tight junction proteins (TJPs) such as ZO-1, OCLN, CLDN-1, subjacent adherens junctions, and desmosomes, which form a physical barrier that separates the intestinal contents from the underlying tissues [10]. Similarly, the TJPs involved in BBB composition were ZO-1, OCLN, and CLDN-5, which maintained structural permeability as well [11]. Catalyzed by the transcription factors Atoh1 and SPDEF, intestinal epithelial stem cells at the intestinal crypts differentiate into cup cells that secrete mucins (MUCs) [12]. Short-chain fatty acids (SCFAs) and metabolites of intestinal flora, can combine with GPR43 (Ffar2) located on the surface of IECs, activate the mTOR signaling pathway, and promote Paneth cells to produce antimicrobial peptides (AMPs) [13], and MUCs are seen as chemical barriers [14]. In addition, intestinal endotoxins that cross the MUC layer prompt the conversion of B lymphocytes in the lamina propria into plasma cells that secrete SIgA, initiating humoral immunity [15]. The intestinal resident CX3CR1+ macrophage population produces interleukin 10 (IL-10), which inhibits the secretion of inflammatory factors by colonic T cells and increases the Treg cell ratio, regulating cellular immune homeostasis [16, 17]. Studies have confirmed that proteins and amino acids in the duodenum induce the secretion of cholecystokinin (CCK), an enteric neuropeptide that acts on VN sensory fibers under the intestinal epithelium to regulate gastrointestinal motility and transmits satiety signals to the brain [18, 19]. These previous findings support the idea that the intestinal mucosa has complex physiological functions and that there is an interaction with the CNS.

Zhongying Zhou, a master of Chinese medicine with more than 70 years of clinical experience, established an LTP formula to treat AICH with obvious effects [20]. The decoction significantly enhances gastrointestinal motility while promoting the recovery of neurological function in AICH [21]. LTP is based on the classic recipe of Xijiao Dihuang decoction from the ancient Chinese medical book “Qian Jin Yao Fang,” with some innovations. LTP consists of eight crude drugs such as Rhei Radix et Rhizoma, Bubali Cornu, Rehmanniae Radix, Paeoniae Radix Rubra, Moutan Cortex, Acori Tatarinowii Rhizoma, Pheretima, and Notoginseng Radix et Rhizoma; among them, Bubali Cornu was substituted for Rhinoceri Asiatici Cornu to comply with the Endangered Species Protection Act [22]. According to traditional Chinese medicine (TCM), the main pathological factors in the acute phase of ICH are blood stasis and fire-heat, categorized as an excess syndrome by syndrome differentiation. These generalizations are based on the nature of symptoms and signs. The formula was for stasis-heat pathology. Rhubarb, the king drug, is mainly used for “purging fu-organs to eliminate heat,” treating excess syndrome with purgation, while the remaining drugs exert an effect of “cooling the blood and dissipating the blood stasis.” Briefly, LTP not only promotes the dissipation of the stasis-heat pathogen at the lesion but also innovatively improves intestinal dynamics, promotes metabolic circulation, and accelerates the clearance of pathogenic factors in the brain through intestinal function. Interestingly, this concept of organic wholeness coincides with the brain-gut axis theory. In our preliminary studies, we found that LTP had a significant reparative effect on intestinal mucosal injury by detecting the levels of lipopolysaccharide (LPS), diamine oxidase (DAO), and D-lactic acid (DLA) in the serum of the AICH rats [23]. Among other things, excess LPS in blood circulation also exacerbates BBB damage [24]. In the case of BBB damage, harmful substances of enteric origin can reach the brain via the blood and aggravate the condition.

Based on the previous results, in the current study, we aimed to explore the following: (1) whether LTPs could improve neurological deficits, brain edema, and BBB disruption in the AICH rats; (2) whether LTPs could enhance IMB functions such as epithelial permeability, MUCs, immune factors, and GPR43 secretion, and maintain T-cell homeostasis and VN activity; and (3) whether there is a more efficacious pathway for LTPs in the treatment of AICH than simply supplementing the intestinal flora by comparing the effects with those of the probiotic group.

## 2. Materials and Methods

### 2.1. Biochemical Materials

The following primary antibodies were used for western blotting: rabbit anti-ZO-1 (Thermo Fisher Scientific, 40–2200, 1 : 1000), OCLN (Abcam, ab167161, 1 : 1000), CLDN-5 (ABclonal, A10207, 1 : 1000), CLDN-1 (Thermo Fisher Scientific, 71–7800, 1 : 1000), SIgA (Bioss Biotech, bs-0645R, 1 : 1000), MUC2 (Boster Biotech, BM5029, 1 : 1000), GPR43 (Bioss Biotech, bs-13536R, 1 : 1000), and a *β*-actin antibody (ABclonal, ac026, 1 : 10000). The secondary antibody was goat anti-rabbit IgG H&L/HRP (Abcam, ab6712, 1 : 5000). The following antibodies were used for flow cytometry: CD3-FITC (11-0030-82), CD8a-PerCP-eFluor 710 (46-0084-82), IL-17A-PE (12-7177-81), CD4-FITC (11-0040-82), CD25-PerCP-eFluor 710 (46-0390-82), and FoxP3-PE (12-5773-82). These antibodies were all purchased from eBioscience (San Diego, CA, USA). A Fix&Perm Buffer kit (421403) was purchased from BioLegend (San Diego, CA, USA). Ionomycin (I139530) and Evans Blue (E104208) were purchased from Aladdin (Los Angeles, CA, USA). Phorbol 12-myristate 13-acetate (PMA, MB5349-1), formamide (MB2593), and brefeldin A (MB3357-1) were purchased from MeilunBio (Dalian, China). A lymphocyte separation medium kit (LTS1083) was obtained from TBD Science (Tianjin, China). PrimeScript RT Master Mix (Perfect Real Time) and SYBR Premix Ex Taq were obtained from Takara Bio (Osaka, Japan). All other chemicals were purchased from Bio-Rad Laboratories, Inc. (Hercules, CA, USA) and Beyotime Biotech Co., Ltd. (Shanghai, China).

### 2.2. Preparation of LTPs

LTP consists of eight crude drugs: rhubarb (10 g), buffalo horn (30 g), raw Rehmannia (20 g), red peony root (15 g), tree peony bark (10 g), Acorus calamus (10 g), earthworm (10 g), and *Panax notoginseng* (10 g) (Table 1). The aforementioned herbs were concentrated and decocted for adult humans by Nanjing Hospital of Chinese Medicine Affiliated with the Nanjing University of Chinese Medicine, containing 1 g crude medicine per mL. In brief, 115 g crude LTP was soaked in 1000 mL pure water for 2 h, and buffalo horn was first decocted for 30 min. Then, other medicinal materials were added to a boil, filtered, repeated twice, and mixed.

### 2.3. LC‒MS/MS Analysis of LTP Components

The qualitative analysis of compounds in the aqueous extract of LTP was performed on an ultrahigh-performance liquid chromatography (UPLC) system (Model: 1290, Agilent, Santa Clara, CA, USA) with an ACQUITY UPLC BEH C_18_ column (1.7 *μ*m 2.1 × 100 mm, Waters, Milford, MA, USA). The flow rate was set at 0.4 mL/min and the sample injection volume was set at 5 *μ*L. The mobile phase consisted of 0.1% formic acid in water (A) and 0.1% formic acid in acetonitrile (B). Acetonitrile and formic acid were purchased from CNW Technologies (Dusseldorf, Germany). The multistep linear elution gradient program was as follows: 0–3.5 min, 95–85% A; 3.5–6 min, 85–70% A; 6–6.5 min, 70–70% A; 6.5–12 min, 70–30% A; 12–12.5 min, 30–30% A; 12.5–18 min, 30–0% A; 18–25 min, 0–0% A; 25–26 min, 0–95% A; and 26–30 min, 95–95% A. A Q Exactive Focus mass spectrometer (Thermo Fisher Scientific, Waltham, MA, USA) coupled with Xcalibur software was employed to obtain the MS and MS/MS data based on the IDA acquisition mode. During each acquisition cycle, the mass range was from 100 to 1500, the top three of every cycle were screened and the corresponding MS/MS data were further acquired. Sheath gas flow rate: 45 Arb, aux gas flow rate: 15 Arb, capillary temperature: 400°C, full ms resolution: 70000, MS/MS resolution: 17500, collision energy: 15/30/45 in NCE mode, and spray voltage: 4.0 kV (positive) or −3.6 kV (negative).

### 2.4. Preparation of Probiotic Solution

The probiotic formula is a live combined bifidobacterium, lactobacillus, and enterococcus capsules (Lot No.: 22120210323) purchased from Shanghai Shangyao Xinyi Pharmaceutical Factory. The formula was prepared by dissolving 17.5 mg of initial crude drug per mL of solution in saline at 4°C.

### 2.5. AICH Rat Model Establishment and Treatment

Thirty male specific-pathogen-free (SPF) Sprague‒Dawley (SD) rats (weighing 200 ± 20 g) were produced by Hangzhou Medical College (License No.: SCXK 2019-0002, Hangzhou, China). Rats were housed at the Experimental Animal Center of the Nanjing University of Chinese Medicine at 20.0 ± 1.0°C and 50.0 ± 10% humidity under a 12 h light-dark cycle. This experiment was approved by the Experimental Animal Ethics Committee of Nanjing University of Chinese Medicine (Application No. 202105A029). Apart from the normal rats (blank group) and sham-operated rats (sham group), the AICH rat models were established as we previously reported [25]. In brief, the rats were preoperatively fasted and anesthetized by inhalation of 1% halothane with an Animal Microanesthesia Ventilator (Model: R540, RWD Life Science and Technology Co., Shenzhen, China). Then, the right caudate nucleus was located by a Rat Brain Stereotactic Instrument (Model: 68005, RWD Life Science and Technology Co., Shenzhen, China) (3 mm to the right from fontanel). After cranial drilling, 50 *μ*L autologous caudate arterial blood was slowly injected with a micropump combined injection needle at a depth of 6 mm. After the blood injection, the needle was left for 10 minutes, and then, the needle was withdrawn by 2 mm. The needle was left for another 5 minutes, and the needle was completely withdrawn. The sham group was treated similarly, except that the right caudate nucleus was inserted with an empty needle but no blood was injected. Bone wax was used to seal the pinhole and suture the surgical incision. The neurological function score of the AICH rats was assigned according to Longa's score on a five-point scale [26], and the models were successfully built for those with a score of 1–3. Then, according to the random number table method, the AICH rats were randomly divided into the model group, LTP group, and probiotics group, with 6 rats per group.

In addition to the blank group, the other 24 rats (6 in each group) were treated according to the following methods: the sham and model groups were intragastrically (IG) administered normal saline (10 mL/kg), the LTP group was given an LTP decoction (dose: 10 g/kg, IG), and the probiotics group was given probiotics solution (dose: 0.175 g/kg, IG). This dose of LTP was confirmed to be more effective in early dose-gradient pharmacological experiments [27, 28]. The frequency of treatment was once a day for a period of 3 days. In the preliminary study, we observed the positive effects of LTP at 24 h, 72 h, and 120 h after the AICH rat model was established; and we found that the results began to be significant at 72 h, the effect remained stable until 120 h that we have observed [29].

### 2.6. Neurological Function Score

According to Longa's scoring method [26], before and after treatment, the degree of neurological deficits in the AICH rats was assessed and the scores were recorded. The average score was calculated by two professional researchers.

### 2.7. Brain Water Content

After the rats were sacrificed by anesthesia overdose, the whole brain was removed immediately by decapitation, and then, the olfactory bulb junction, cerebellum, and low brainstem were harvested. The surface blood stains were rinsed with saline, and the right cerebral hemisphere containing the hematoma was separated. The water stains were dried with disposable absorbent paper and immediately placed in a dry Petri dish and weighed on a microbalance, i.e., wet weight. The right cerebral hemisphere was held in tin foil, dried in a constant temperature oscillating incubator at 60°C for 24 h, and then weighed, i.e., dry weight. Brain water content (%) =  (wet weight − dry weight)/wet weight × 100%.

### 2.8. Evans Blue (EB) Staining

After the rats were anesthetized, 4% EB solution (dose: 2 mL/kg) was injected into the femoral vein, and the terminal skin and eyes of the rats gradually turned blue during the injection. One hour after the injection, the thoracic cavity of the rats was opened, and normal saline (300 ± 20 mL) was injected through the left ventricle while the right atrium was cut open, and perfusion was stopped when the fluid flowing from the right atrium became clear. Following cardiac perfusion, the right cerebral hemisphere of the rats was stripped and the surface was rinsed well with cold normal saline. Water stains on the surface were dried with absorbent paper and the brain was weighed. Then, formamide (dose: 10 mL/g) was added and dissolved in a constant temperature shock incubator at 60°C for 24 h. The EB standard solution was prepared by the gradient titration method with a suitable concentration and added to the NO. 1–6 centrifuge tubes, which were similarly dissolved as the samples to be tested. The supernatants of the EB standard and untested samples were extracted after centrifugation (2000 rpm × 20 min) and added to a 96-well plate at a volume of 200 *μ*L per well. The optical density (OD) value was measured at 632 nm using an EnSpire Multimode Plate Reader (PerkinElmer, Waltham, MA, USA). A regression curve was drawn based on the OD value of the standard substance, the concentration of EB in the sample was calculated by the curve equation, and the volume of EB infiltration in the rat brain tissue was obtained. The value of EB infiltration (*μ*g·g^−1^) = Concentration of EB in the sample (*μ*g·mL^−1^) × volume of formamide (mL)/wet weight of brain (g).

### 2.9. Reverse Transcription-Polymerase Chain Reaction (RT-PCR)

Total RNA was extracted from the brain hematoma tissues, the purity and concentration were determined, and was then reverse transcribed with PrimeScript RT Master Mix according to the manufacturer's protocol. A real-time PCR was operated following the two-step quantitative RT-PCR method with SYBR Mixture. The amplification of genes was analyzed through a 7500 real-time PCR system (ABI, Waltham, MA, USA). Primer sequences are as follows:

  TNF-*α* Forward: 5′-ATGGTCACCCTCAGATCAGC-3′
  TNF-*α* Reverse: 5′-TTGACCGCTGAAGAGACCCT-3′
  IL-1*β* Forward: 5′-CAGCAGCATCTCGACAAGAG-3′
  IL-1*β* Reverse: 5′-AAAGAAGGTGCTTGGGTCCT-3′
  GAPDH Forward: 5′-AAGGGCTCATGACCACAGTC-3′
  GAPDH Reverse: 5′-GGATGCAGGGATGATGTTCT-3′

All primers were designed and synthesized by Shenggong Bio (Shanghai, China).

### 2.10. Western Blot (WB) Analysis

Cold RIPA Lysis Buffer (P0013B, Beyotime) with protease inhibitors (P1005, Beyotime) was used to extract the total protein from the tissue around the brain hematoma (Radius: 1 mm) and the distal colon (Below cecum: 7–10 cm). The protein concentration was quantified by a BCA kit (P0010, Beyotime), normalized, mixed with Laemmli Sample Buffer (161-0747, Bio-Rad), and heated in a 95°C water bath for 5 minutes. Ten micrograms of protein samples from each group were electrophoresed through a 10% SDS‒PAGE gel, transferred onto a polyvinylidene fluoride (PVDF) membrane (Merck Millipore Ltd., Darmstadt, Germany), and blocked with TBST (containing 5% nonfat dry milk) for 2 h at room temperature. After sequential incubation with appropriate primary antibodies and secondary antibodies conjugated to HRP, the positive western blots were detected with an ImageQuant LAS4000 mini biomolecular imager system (GE Healthcare Life Sciences, Fairfield, CT, USA) by chemiluminescence using an ECL kit (P0018S, Beyotime). The internal reference for expression levels was *β*-actin.

### 2.11. Flow Cytometry (FCM) Analysis

Peripheral blood mononuclear cells (PBMCs) from rats were prepared with lymphocyte separation medium by density gradient centrifugation. The cells in the PBMC suspension were counted and the concentration was adjusted to 1 × 10^6^/mL. A mixture of ionomycin [final concentration (FC): 1 *μ*g/mL], PMA (FC: 100 ng/mL), and brefeldin A (FC: 10 *μ*g/mL) was added to the appropriate volume of cell suspension sample and then placed in a 5% CO_2_ incubator at 37°C for 6 h to stimulate T cells to secrete cytokine interleukin-17A (IL-17A). The cultured cell samples in a final volume of 100 *μ*L were stained with CD3-FITC (0.25 *μ*g) and CD8a-PerCP-eFluor 710 (0.03 *μ*g) antibodies. Cells were then fixed, permeabilized, and stained with PE-labeled IL-17A (0.125 *μ*g) antibody according to the manufacturer's instructions, washed using PBS by centrifugation (1500 rpm × 10 min), and resuspended in a volume of 1 mL. Ultimately, the detection of samples was performed by the Cytomics FC 500 MPL Flow Cytometer, (Beckman Coulter, Inc., Brea, CA, USA). In addition, an additional equal volume of cell samples was stained with CD4-FITC (0.125 *μ*g) and CD25-PerCP-eFluor 710 (0.03 *μ*g) antibodies before being fixed and permeabilized, and then with Foxp3-PE (0.5 *μ*g) antibody.

### 2.12. Vagus Nerve (VN) Discharge

Preoperative fasting was performed for all rats. After anesthesia, an incision of approximately 2.5 cm was made on the left side of the subxiphoid process, and the peritoneum and muscular layer were peeled away to expose the VN gastric branch at the wall of the pyloric canal under the microscope, which was separated by approximately 1 cm with a glass minute needle. The neuroprotective electrodes of the bioelectrical signal acquisition and processing system (Model: RM6240BD, Chengdu Instrument Factory, China) were connected to the distal and proximal ends of the VN fiber to collect the discharge signal waveform. Meanwhile, drops of warm paraffin oil were added to moisten the area to prevent nerve and tissue drying. Interference was eliminated through grounding. The parameters are as follows: acquisition frequency 20 kHz, scanning speed 100 ms/div, sensitivity 50 *μ*V, time constant 0.001 s, and filtering intensity 3 KHz. The recording was started after the VN discharge signal waveform was stable, and lasted for 5 minutes. The rate of VN discharge (times/minute) = Total number (times)/Total time (minute). The amplitude (*μ*V) could be measured with the system as well.

### 2.13. Statistical Analysis

Data are expressed as the mean ± SD values. Statistical analysis was performed in SPSS version 25.0 software. Except for the comparison of Longa's score values before and after treatment in the same group of rats using a *t*-test or nonparametric test, one-way analysis of variance (ANOVA) tests were used for comparisons between the groups in the rest of the data followed by the least significant difference (LSD) test for multiple comparisons. *p* < 0.05 was considered a statistically significant difference.

## 3. Results

### 3.1. Qualitative Analysis of Components in the Aqueous Extract of LTP

**Figure 1:**
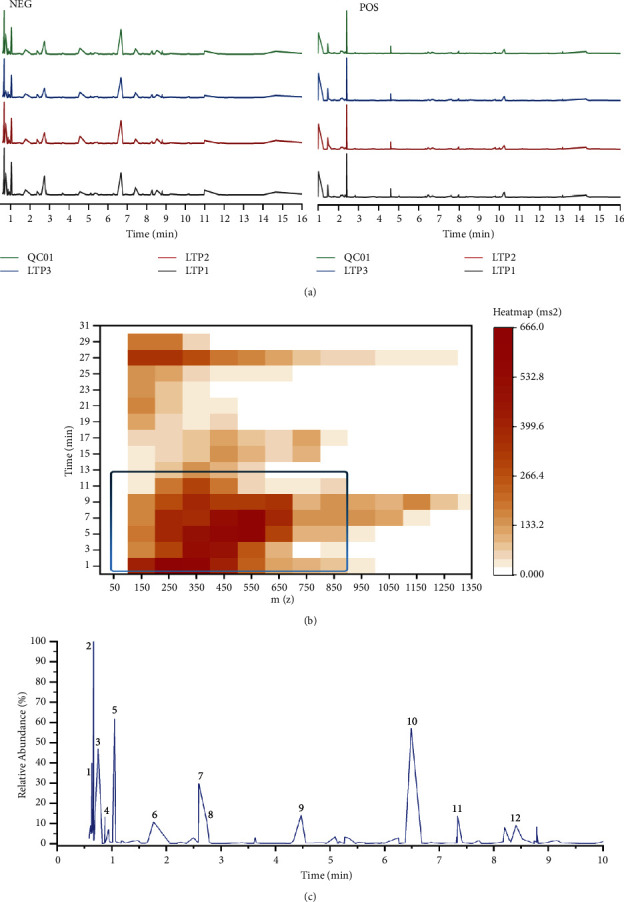
Quality control and main components identification of LTPs: (a) fingerprints of the LTPs and QC samples, (i) negative ion mode and (ii) positive ion mode, (b) MS2 heatmap of LTP sample, and (c) base peak chromatogram of LTP in negative ion mode.

For the quality evaluation of different batches of drugs, the components in the aqueous extract of LTP samples were analyzed by a UPLC-QE-MS method. A total ion chromatogram of LTP is shown in supplementary figure s1. The ion fragment peaks of unknown metabolites that were hard to define in each group of samples were discarded through searching and matching with the laboratory's self-built database, integrated public database, AI database, and metDNA. 157 compounds were included in subsequent analyses. Comparing the superimposed fingerprints of three batches of LTP samples and a quality control (QC) sample, there is no obvious difference. Thus, it was confirmed that the quality of the drugs used in our study is reliable and representative, and the metabolites of LTP could have a higher detection rate in the negative ion mode (Figure 1(a)). As shown in the secondary mass spectrometric heatmap (Figure 1(b)) of total metabolites, the retention time of MS2 fragments in LTP components was mainly concentrated in the range of 0–10 minutes. The analysis of ion characteristics from the heatmap was consistent with the base peak chromatogram (Figure 1(c)), and 12 active ingredients with relatively high abundance were identified. The detailed information on these components was presented in supplementary table s2. The names of the main compounds are as follows: (1) d-gluconic acid, (2) citrate, (3) d-(+)-malic acid, (4) l-pyroglutamic acid, (5) gallic acid, (6) leonuride, (7) epicatechin, (8) oxypaeoniflorin, (9) dencichine, (10) ginsenoside Rf, (11) apigenin-7-O-glucoside, and (12) ginsenoside Rb1.

### 3.2. LTP Alleviated Brain Damage in AICH Rats

**Figure 2:**
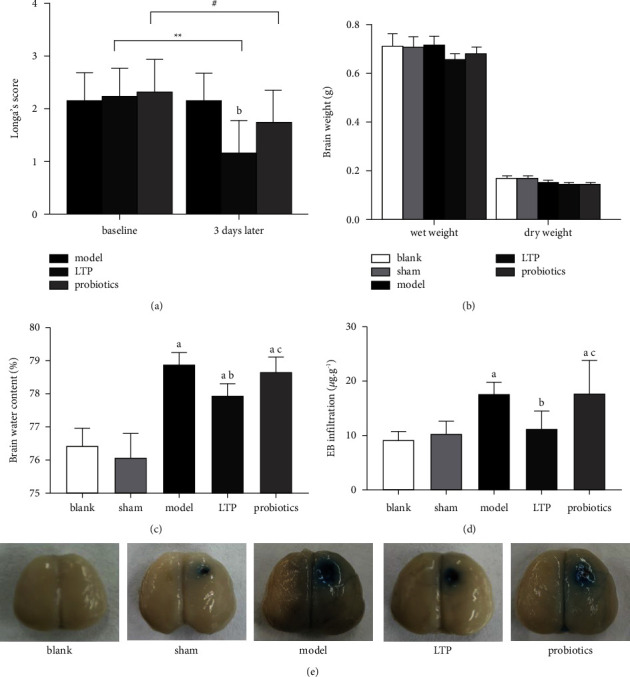
Effect of LTP on brain damage in the AICH rats: (a) Longa's score before and after treatment, (b) brain weight, (c) brain water content, (d) volume of EB infiltration in brain tissue, and (e) appearance of EB staining in rat brains. ^*∗∗*^*p* < 0.01and ^#^*p* < 0.05 vs. before treatment. ^a^*p* < 0.01 vs. the blank and sham groups. ^b^*p* < 0.01 vs. the model group. ^c^*p* < 0.05 vs. the LTP group (*n* = 6).

Longa's scores of rats in both the blank and sham groups were 0. Longa's scores of the AICH rats were not significantly different between the model, LTP, and probiotics groups in the baseline period before treatment. After 3 d of treatment, the scores were significantly lower in the LTP group than in the model group (*p* < 0.01), and there was no significant difference between the probiotics and model groups. Longa's scores were significantly lower in the LTP and probiotics groups after treatment than before treatment (*p* < 0.01, *p* < 0.05), and there was no significant difference before and after treatment in the model group (Figure 2(a)). The values of brain wet weight and dry weight of rats in each group are shown in Figure 2(b). The brain water contents in the model, LTP, and probiotics groups were significantly higher than those in the blank and sham groups (*p* < 0.01), and those of the model group increased the most. Compared with the model group, the LTP group was significantly lower (*p* < 0.01), and the probiotics group was higher than those in the LTP group (*p* < 0.05) (Figure 2(c)). Compared with the blank and sham groups, the volumes of EB infiltration in brain tissue were significantly increased in the model and probiotics groups (*p* < 0.01), and significantly decreased in the LTP group when compared with the model group (*p* < 0.01), with no significant difference between the probiotics and model groups. The volumes of the probiotics group were higher than those of the LTP group (*p* < 0.05) (Figures 2(d) and 2(e)).

### 3.3. LTP Attenuated the Neuroinflammation in the Brain of AICH Rats

**Figure 3:**
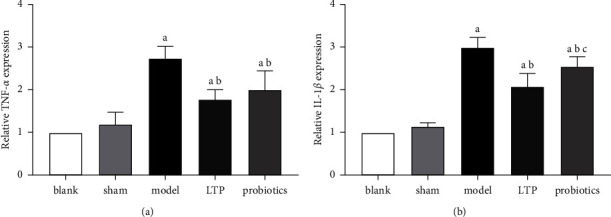
Effect of LTP on the relative expression of proinflammatory factors in the brain tissue of AICH rats: (a) relative TNF-*α* expression, and (b) relative IL-1*β* expression. ^a^*p* < 0.05 vs. the blank and sham groups. ^b^*p* < 0.05 vs. the model group. ^c^*p* < 0.05 vs. the LTP group (*n* = 3).

Compared with the blank and sham groups, TNF-*α* and IL-1*β* mRNA expression in the brain tissue was significantly upregulated in the model, LTP, and probiotics groups (*p* < 0.05). The aforementioned two inflammatory factors expression levels were significantly downregulated in the LTP and probiotics group compared with the model group (*p* < 0.05), and there was no significant difference between the probiotics and LTP groups in TNF-*α* relative expression. The level of IL-1*β* mRNA expression in the probiotics group was higher than that in the LTP group (*p* < 0.05) (Figures 3(a) and 3(b)).

### 3.4. LTP Repaired the BBB Permeability in AICH Rats

**Figure 4:**
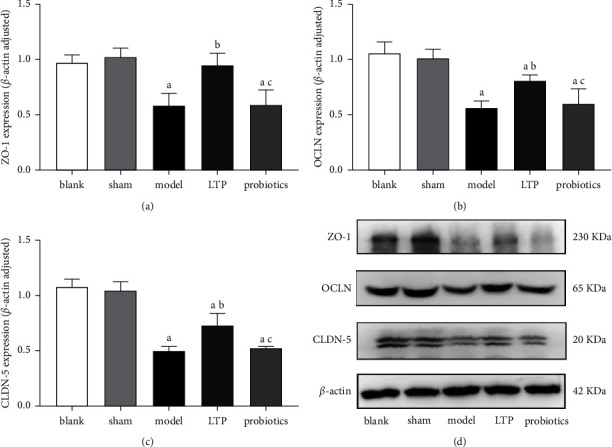
Effect of LTP on the expression of TJPs in the brain tissue of the AICH rats: (a) ZO-1 expression, (b) OCLN expression, (c) CLDN-5 expression, and (d) WB electrophoretograms of the abovementioned TJPs in the brain tissue samples. ^a^*p* < 0.01 vs. the blank and sham groups. ^b^*p* < 0.01 vs. the model group. ^c^*p* < 0.01 vs. the LTP group (*n* = 3).

Compared with the blank and sham groups, OCLN and CLDN-5 protein expression in the brain tissue was significantly downregulated in the model, LTP, and probiotics groups (*p* < 0.01), and ZO-1 protein expression was only downregulated in the model and probiotics groups (*p* < 0.01). The aforementioned three TJP levels were significantly upregulated in the LTP group compared with the model group (*p* < 0.01), and there was no significant difference between the probiotics and model groups, with the levels in the probiotics group being lower than those in the LTP group (*p* < 0.01) (Figures 4(a)–4(d)).

### 3.5. LTP Repaired Epithelial Permeability in the Distal Colon of AICH Rats

**Figure 5:**
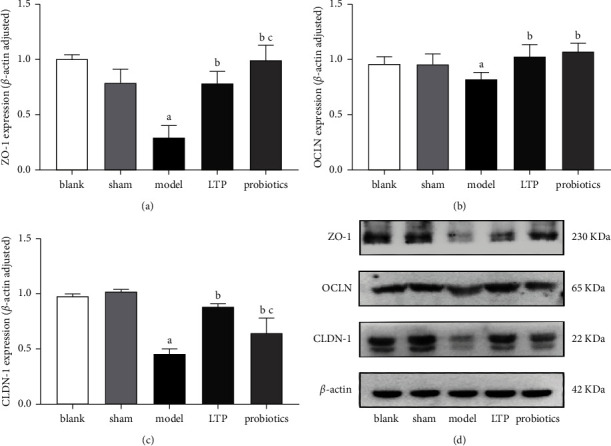
Effect of LTP on the expression of TJPs in the colon tissue of the AICH rats: (a) ZO-1 expression, (b) OCLN expression, (c) CLDN-1 expression, and (d) WB electrophoretograms of the abovementioned TJPs in colon tissue samples. ^a^*p* < 0.05 vs. the blank and sham groups. ^b^*p* < 0.01 vs. the model group. ^c^*p* < 0.05 vs. the LTP group (*n* = 3).

Compared with the blank and sham groups, the ZO-1, OCLN, and CLDN-1 protein expression levels in the colon tissue were significantly downregulated in the model group (*p* < 0.05), and those of the LTP and probiotics groups were significantly upregulated when compared with the model group (*p* < 0.01), CLDN-1 levels were significantly lower and ZO-1 expression was significantly higher in the probiotics group compared to the LTP group (*p* < 0.05), and there was no significant difference in OCLN expression between the two groups (Figures 5(a)–5(d)).

### 3.6. LTP Promoted the Secretion of MUC2, SIgA, and GPR43 in the Colon of AICH Rats

**Figure 6:**
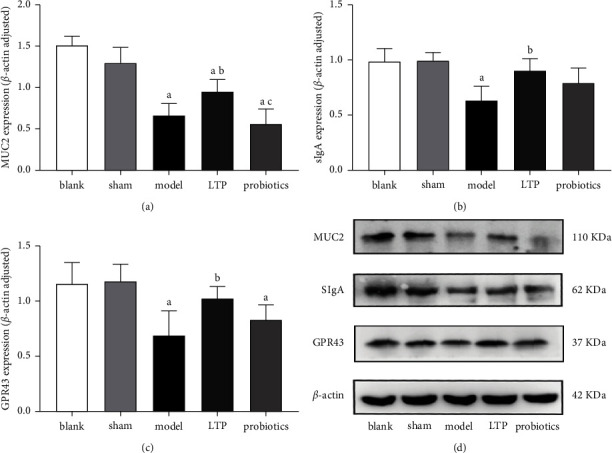
Effect of LTP on MUC2, SIgA, and GPR43 expression in the colon tissue of AICH rats: (a) MUC2 expression, (b) SIgA expression, (c) GPR43 expression, and (d) WB electrophoretograms of MUC2, SIgA, and GPR43 in the colon tissue samples. ^a^*p* < 0.05 vs. the blank and sham groups. ^b^*p* < 0.05 vs. the model group. ^c^*p* < 0.05 vs. the LTP group (*n* = 3).

MUC2 protein expression was significantly downregulated in the model, LTP, and probiotics groups compared with the blank and sham groups (*p* < 0.05), and the expression in the LTP group was significantly upregulated compared with the model group (*p* < 0.05). There was no significant difference between the probiotics and model groups, and the expression in the probiotics group was lower than that in the LTP group (*p* < 0.05) (Figures 6(a) and 6(d)). Compared with the blank and sham groups, SIgA expression was significantly downregulated in the model group (*p* < 0.05), and that of the LTP group was significantly upregulated when compared with the model group (*p* < 0.05). There was no significant difference between the probiotics and model groups (Figures 6(b) and 6(d)). Compared with the blank and sham groups, GPR43 expression was significantly downregulated in the model and probiotics groups (*p* < 0.05), and that in the LTP group was significantly upregulated when compared with the model group (*p* < 0.05). There was no significant difference between the probiotics and model groups (Figures 6(c) and 6(d)).

### 3.7. LTP Maintained the Balance of Th17/Treg Cell in the Peripheral Blood of AICH Rats

**Figure 7:**
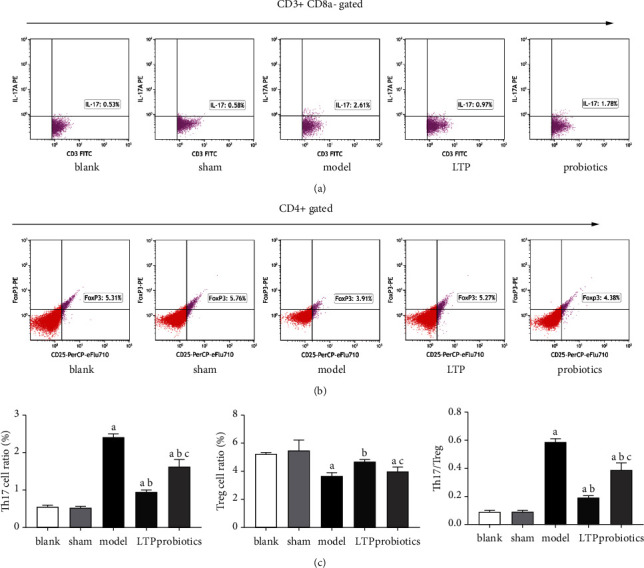
FCM scatter plot of Th17 and Treg cells in peripheral blood: (a) CD3+ CD8a− IL17A + Th17 cells, (b) CD4+ CD25+ Foxp3+ Treg cells, and (c) the ratio of (A) Th17, (B) Treg cells, and (C) Th17/Treg in peripheral blood of each group of rats. ^a^*p* < 0.01 vs. the blank and sham groups. ^b^*p* < 0.01 vs. the model group. ^c^*p* < 0.05 vs. the LTP group (*n* = 3).

Compared with the blank and sham groups, the ratios of the Th17 cells in peripheral blood of the model, LTP, and probiotics groups were significantly higher (*p* < 0.01) and significantly lower in the LTP and probiotics groups when compared with the model group (*p* < 0.01), and those of the probiotics group were higher than those in the LTP group (*p* < 0.05) (Figures 7(a) and 7(c) A). Compared with the blank and sham groups, the Treg cell ratios were significantly lower in the model and probiotics groups (*p* < 0.01) and significantly higher in the LTP group when compared with the model group (*p* < 0.01), and those of the probiotics group were lower than those in the LTP group (*p* < 0.05) (Figures 7(b) and 7(c) B). Compared with the blank and sham groups, the values of Th17/Treg in the model, LTP, and probiotics groups were significantly higher (*p* < 0.01), and significantly lower in the LTP and probiotics groups. When compared with the model group (*p* < 0.01), those of the probiotics group were higher than in the LTP group (*p* < 0.05) (Figure 7(c) C).

### 3.8. LTP Regulated the Discharge of the VN in the Duodenum of AICH Rats

**Figure 8:**
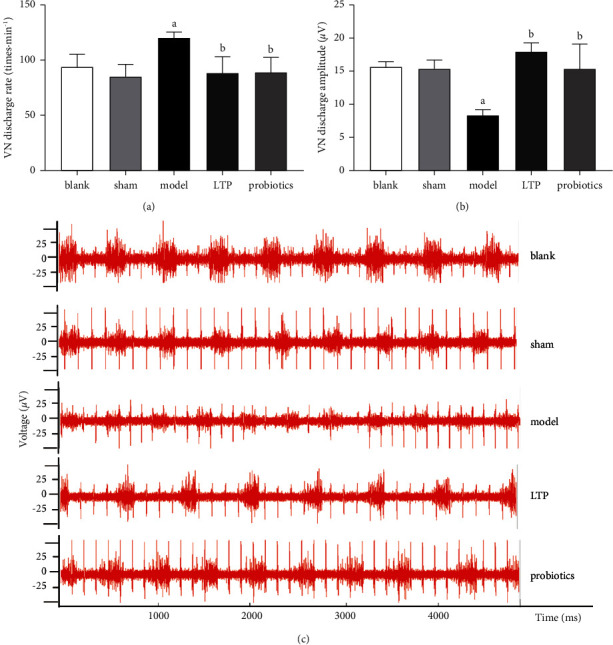
Effect of LTP on the discharge of VN in the duodenum of AICH rats: (a) VN discharge rate, (b) VN discharge amplitude, and (c) waveform of VN discharge in a 5 seconds period. ^a^*p* < 0.01 vs. the blank and sham groups. ^b^*p* < 0.01 vs. the model group (*n* = 6).

Compared with the blank and sham groups, the VN discharge rates in the model group were significantly faster and the amplitudes were significantly lower (*p* < 0.01). When compared with the model group, the discharge rates were significantly slower and the amplitudes were significantly higher in the LTP and probiotic groups (*p* < 0.01), with no significant difference between the LTP and probiotic groups (Figures 8(a)–8(c)).

## 4. Discussion

Increasing research on neurological disorders has focused attention on the brain-gut axis. AICH is often associated with intestinal system complications such as disturbance of intestinal flora, impaired fecal excretion, and stress ulcers. Hang et al. found that [30] severe histopathological changes occurred in the intestinal mucosa at 3 h after the onset of traumatic brain injury (TBI), with further apoptosis of IECs and disruption of intercellular tight junction structures, intestinal mucosal permeability also increased. In addition, Yu et al. used [31] healthy fecal microbiome transplantation (FMT) to treat ICH mice, which can restore the homeostasis of the intestinal environment, protect IMB function, and promote the transfer of immune lymphocytes from the intestine to the brain, thus reducing neuroinflammatory damage and improving the prognosis of ICH. However, the specific pathophysiological link between intestinal microecology and ICH is yet to be revealed. Furthermore, the intestinal mucosa, an important carrier of intestinal microecology, has been shown to form a natural protective barrier through IECs, TJPs, the MUC layer, immune cells, and the enteric nervous system (ENS), reducing the invasion of pathogenic bacteria and inflammatory factors [32, 33]. With the lack of effective drugs against AICH, the use of herbal medicine to improve IMB function may be a potential therapeutic option that deserves in-depth investigation.

According to the flavor and efficacy of the herbs used, the compatibility strategy of LTP was aimed at the two pathological factors of blood stasis and fire-heat highlighted in the acute stage of ICH. Blood stasis refers to the stagnation of blood from ruptured cerebral vessels to form a space-occupying effect in the brain, and the resulting local hematoma pressure and thrombin toxicity could cause brain edema. Fire-heat manifested as an inflammatory response in AICH. Our previous study demonstrated that LTPs reduce the activation of toll-like receptor 4 (TLR4) and inhibit inflammatory responses mediated by the nuclear factor kappa-B (NF*κ*B) signaling pathway in AICH rats [34]. Kwon also demonstrated in a rat model of reflux esophagitis that rhubarb components could achieve anti-inflammatory effects by inhibiting the NF*κ*B pathway [35]. Xu et al. found the same antioxidant effect of rhubarb in a rat model of TBI [36]. Our current study data showed that LTPs reduced the degree of neurological deficits, attenuated brain edema, repaired BBB permeability, and attenuated the neuroinflammation in AICH rats, moreover, the repairing effect of LTPs on the BBB yielded consistent results in both EB staining (Figures 2(d) and 2(e)) and WB analysis (Figures 4(a)–4(d)). Gallic acid and epicatechin are abundant in the LTP compositions. Studies have confirmed that [37, 38] gallic acid significantly improves neurological damage in TBI rats, and Cheng's study also showed that epicatechin has a restorative effect on neurological deficits in TBI mice [39]. The antioxidant effect of LTPs was consistent with the expected treatment method of “cooling the blood and dissipating the blood stasis.” Combined with another therapeutic principle “purging fu-organs to eliminate heat” of LTP, the fu-organ mentioned here is the intestine, which is responsible for the excretion of feces. This effect of the monarch drug rhubarb was demonstrated in a recent study, and rhubarb extract could increase the secretion of MUCs by cupular cells and regulate intestinal flora in the colon to promote stool evacuation [40]. To explore the potential mechanisms of this core therapeutic strategy, we focused our subsequent studies on the intestinal system.

The intestine is the largest immune organ in the body, and the colon has a high abundance of flora and exuberant secretory activity; in particular, high levels of MUCs are maintained in the distal colon, which has a protective effect on the intestinal mucosa [41, 42]. The IMB consists of the IEC junction complex and its secretions, intestine-associated immune cells, and the intrinsic intestinal flora. The MUCs secreted by cup cells in the intestinal epithelium adhere to the outermost layer of the intestinal mucosa, which is rich in AMPs and SIgA, preventing pathogenic bacteria from reaching the intestinal epithelium [43]. Moreover, IgA-secreting plasma cells in the intestine can be transferred to the brain and exert a protective effect on the central nervous system [44]. TJPs in the intestinal epithelium act as the basis for the intestinal mechanical barrier and prevent endotoxin and inflammatory factors from entering the bloodstream for infection, while the BBB also relies on TJPs to maintain permeability and avoid harmful substances from invading the brain. The intestinal flora and its metabolites can induce Foxp3 expression via GPR43, increase Treg cell differentiation, and suppress excessive inflammatory responses mediated by Th17 cells, as has been demonstrated by numerous authors [45–47]. GPR43 can also exert immunomodulatory effects by modulating Group 3 innate lymphoid cells (ILC3s) [48]. The results of our experiment confirmed that LTP significantly increased the secretion of MUC2, SIgA, and GPR43 in the colon of the AICH rats, while the probiotics had no significant reparative effect. In addition, by analyzing the expression of ZO-1, OCLN, and CLDN-1, the intestinal mucosal TJPs in each group of rats, we found that both LTP and probiotics had significant reparative effects on intestinal permeability in AICH rats. At the beginning of the manuscript, we introduced that our recent report also proved that both LTP and probiotics can significantly reduce the content of LPS in the serum of AICH rats [23]. Stimulated by LPS, monocytes and macrophages can rapidly release proinflammatory factors, such as TNF- *α* and IL-1 *β*. In the current study, we also found that LTP and probiotics decreased the mRNA level of TNF- *α* and IL-1*β* in the brain tissue of the AICH rats, and the inhibitory effect of LTP on IL-1 *β* is better. Accordingly, the protective effect of LTP on IMB might indirectly prevent intestinal proinflammatory toxins from invading the brain in AICH rats. Although this view of explaining the brain-gut axis theory may only be a possibility, it is consistent with the latest report from Pellegrini et al. [49]. We also performed FCM analysis of lymphocytes in the peripheral blood of each group of rats, showing that both LTPs and probiotics had a regulatory effect on Th17/Treg cell imbalance, but LTPs were superior to probiotics. Wang et al. found that [50] disorders of intestinal flora can cause histological damage to the intestinal mucosa with disruption of tight junctions and increased bacterial translocation in a mouse model. Chen et al. demonstrated that [51] ginsenoside Rb1 could regulate *Lactobacillus helveticus* abundance and *γ*-aminobutyric acid (GABA) receptor expression to exert neuroprotective effects in a cerebral ischemia-reperfusion injury rat model. These findings suggest that intestinal flora have a modulatory effect on both intestinal mucosal permeability and T-cell immunity, which was confirmed in the probiotic group of rats in our study. However, the LTP component could also affect more classes of intestinal strains, produce more abundant MUCs, SIgA, and GPR43, better modulate the immune function of the intestinal mucosa, and suppress the excess inflammatory response, thus exerting a better therapeutic effect than the probiotics.

VN, which passes through the brain and intestine, undergoes signal communication between gut microbes and the brain [52]. The analysis of VN discharge signals in the AICH rat model was valuable for exploring the neuroimmune regulatory pathways of the brain-gut axis. We collected the waveforms of VN discharge signals from the duodenal segment of rats in each group and found that the excitability of the VN in the AICH model rats was suppressed, as evidenced by a significant decrease in amplitude and a compensatory increase in discharge rate. LTP and probiotics could reverse this discharge suppression state of VN to different degrees. Comprehensive analysis showed that the LTPs and probiotics had comparable effects in repairing the epithelial structure and VN conduction function in the intestinal mucosa of the AICH rats. Zhou et al.'s study confirmed that [53] VN stimulation attenuates the disruption of intestinal mucosal tight junctions in endotoxemic mice via a cholinergic anti-inflammatory pathway. Accordingly, the activity of VN fibers attached to the intestinal wall may be interdependent with intestinal epithelial integrity; however, the exact association needs to be elucidated by additional neurophysiological studies.

## 5. Conclusions

Ultimately, we demonstrated that LTPs ameliorate neurological deficits, brain edema, BBB permeability, and neuroinflammation, thereby, slowing the progression of brain hemorrhage in the acute phase. In addition, LTPs could repair intestinal mucosal epithelial structures, promote the secretion of MUC2, SIgA, and GPR43, and regulates the activity of VN in the duodenal segment and the Th17/Treg ratio in peripheral blood, thus enhancing IMB function. Meanwhile, we also found that probiotics had a significant restorative effect on intestinal mucosal epithelial permeability and VN activity. The view that a stable intestinal mucosal function could promote the repair of brain damage is relatively clear through analyzing the connection between the anti-inflammatory effects of LTP and probiotics on the brain-gut axis of the AICH rats. To sum up, in order to explore the main therapeutic approaches that LTP is superior to probiotics in AICH rats, we will focus on the signaling pathway neuroimmune mediated between the gut and the brain in the future, thereby, providing novel ideas for the clinical treatment of AICH. However, the pharmacological effects of LTP components require more in-depth *in vitro* experimental studies.

## Figures and Tables

**Table 1:** Ingredients of LTP.

English names	Latin names	Part used	Quantity (dry) (g)
Rhubarb	Rhei Radix et Rhizoma	Root	10
Buffalo horn	Bubali Cornu	Horn	30
Raw Rehmannia	Rehmanniae Radix	Root	20
Red peony root	Paeoniae Radix Rubra	Root	15
Tree peony bark	Moutan Cortex	Bark	10
*Acorus calamus*	Acori Tatarinowii Rhizoma	Root	10
Earthworm	Pheretima	Carcass	10
*Panax notoginseng*	Notoginseng Radix et Rhizoma	Root	10

## Data Availability

The data used to support the findings of this study are available from the corresponding author upon reasonable request.

## Supplementary Materials

Supplementary Materials (1) Supplementary figure s1: a total ion chromatogram of LTP sample (from part 3.1). (2) Supplementary table s2: the detailed information of LTP main compounds (from part 3.1).
