# Supplementary figures and images for: Liangxue Tongyu Prescription Alleviates Brain Damage in Acute Intracerebral Hemorrhage Rats by Regulating Intestinal Mucosal Barrier Function
Source: Evid Based Complement Alternat Med. 2022 Dec 17;2022:2197763. doi: 10.1155/2022/2197763 (PMC9789913; doi:10.1155/2022/2197763)

(a) NEG

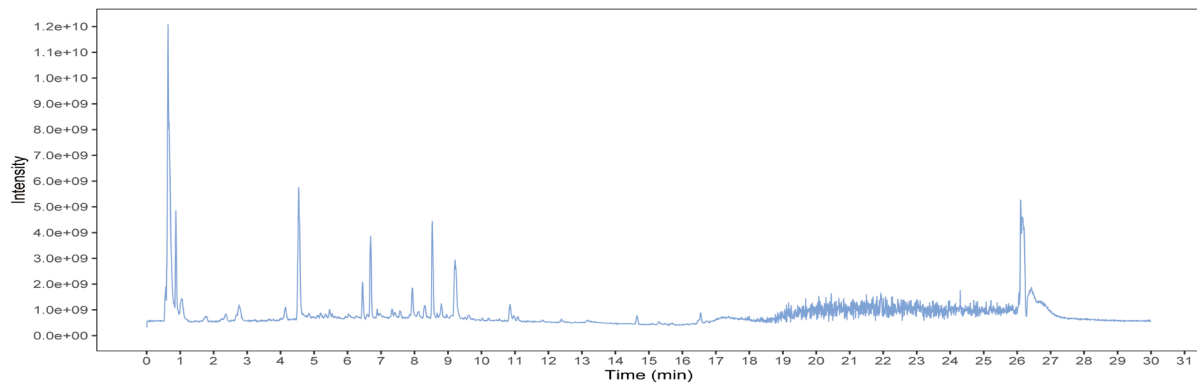

(b) POS

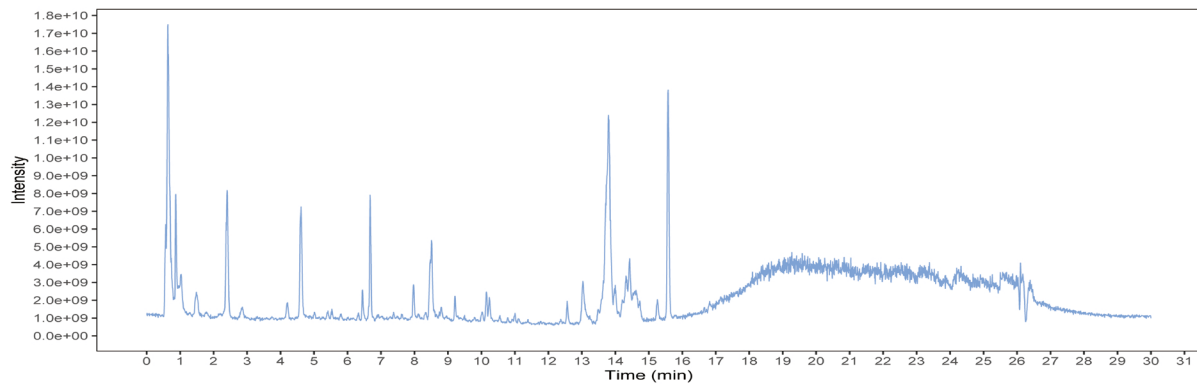

Supplement: Supplementary Materials — (1) Supplementary figure s1: a total ion chromatogram of LTP sample (from part 3.1). (2) Supplementary table s2: the detailed information of LTP main compounds (from part 3.1). [file 2197763.f1.zip › A Supplementary figure s1.pdf]
